# Correction: Exploiting the promiscuity of imatinib

**DOI:** 10.1186/1741-7007-8-82

**Published:** 2010-06-10

**Authors:** Shun J Lee, Jean YJ Wang

**Affiliations:** 1 Department of Medicine and Division of Hematology-Oncology, School of Medicine, University of California, San Diego, La Jolla, CA 92093, USA; 2 Moores Cancer Center, 3855 Health Sciences Drive, University of California, San Diego, La Jolla, CA 92093-0820, USA; 3 Division of Biological Sciences, University of California, San Diego, La Jolla, CA 92093, USA

## 

An error in the PDB code occurred in the article published in the *Journal of Biology *[1]. In the legend to Figure One, instead of 1FW3, the correct PDB code is 3FW1.
